# Supplementary figures and images for: A novel immune-related prognostic signature based on Chemoradiotherapy sensitivity predicts long-term survival in patients with esophageal squamous cell carcinoma
Source: PeerJ. 2023 Aug 18;11:e15839. doi: 10.7717/peerj.15839 (PMC10441524; doi:10.7717/peerj.15839)

# IL-17 SIGNALING PATHWAY

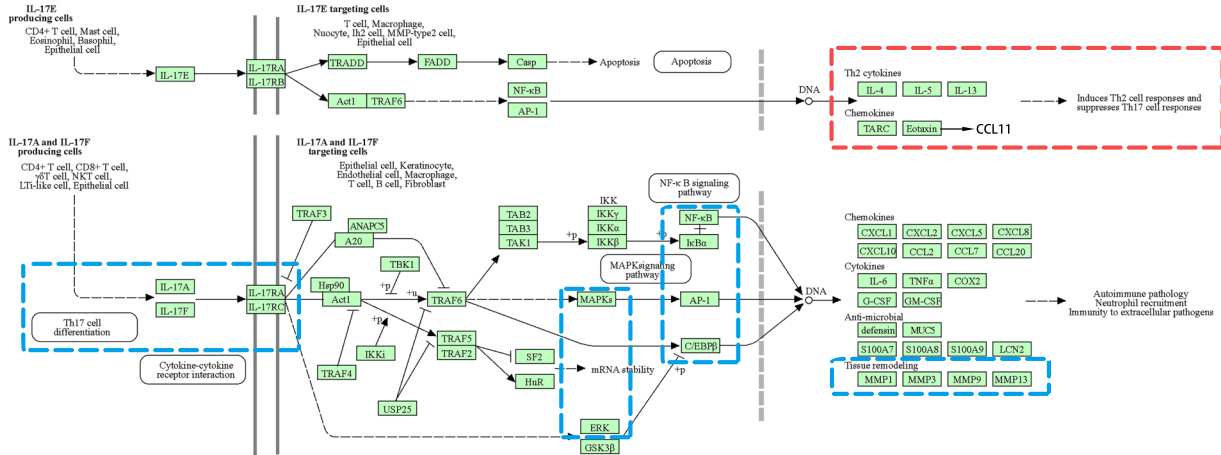

Supplement: Figure S1 — MMP1/3/9/13 are located downstream of IL-17A/F. IL-4/5/13 and CCL11 (Eotaxin) can inhibit Th17 cells and promote a Th2 cell response. [file peerj-11-15839-s001.pdf]

# Inhibition of TH17 differentiation

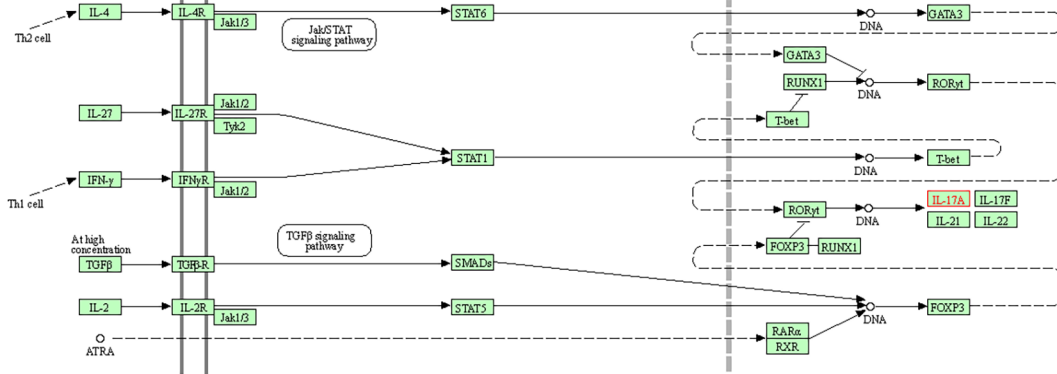

Supplement: Figure S2 — Based on the Th17 cell differentiation pathway, IL-4 could up-regulate GATA3 through the JAK/STAT signaling pathway, blocking RORC DNA expression, thereby reducing IL-17A secretion. [file peerj-11-15839-s002.pdf]

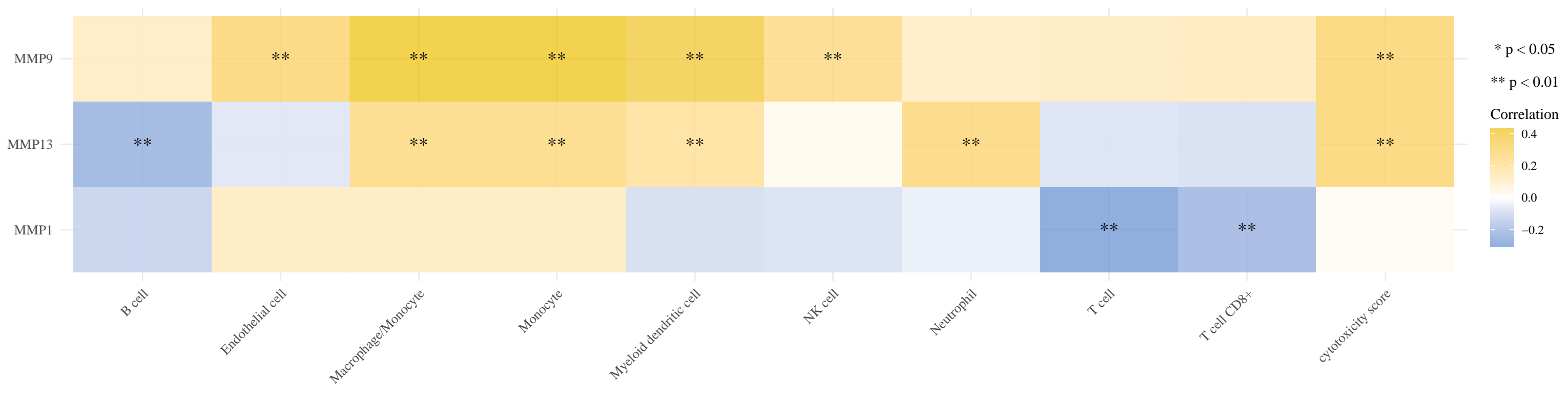

Supplement: Figure S3 — The abscissa represents tumor immune cells, and the ordinate represents genes, where different colors represent correlation coefficients, and darker colors represent stronger correlations between the two. * p < 0.05, ** p < 0.01. [file peerj-11-15839-s003.pdf]

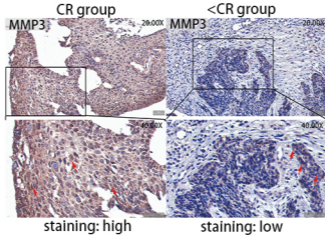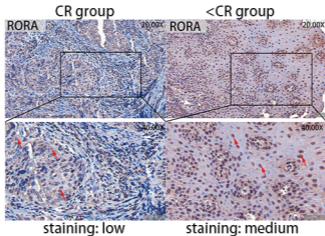

Supplement: Figure S4 — IHC staining was performed to evaluate the level of MMP3 and RORA in cancer tissues. Compared with <CR group, MMP3 protein staining was darker in CR group, but RORA protein staining was lighter. Original magnification: 20× and 40× (inset panels). IHC, immunohistochemistry; CR, complete remission; <CR, CR not achieved. [file peerj-11-15839-s004.pdf]

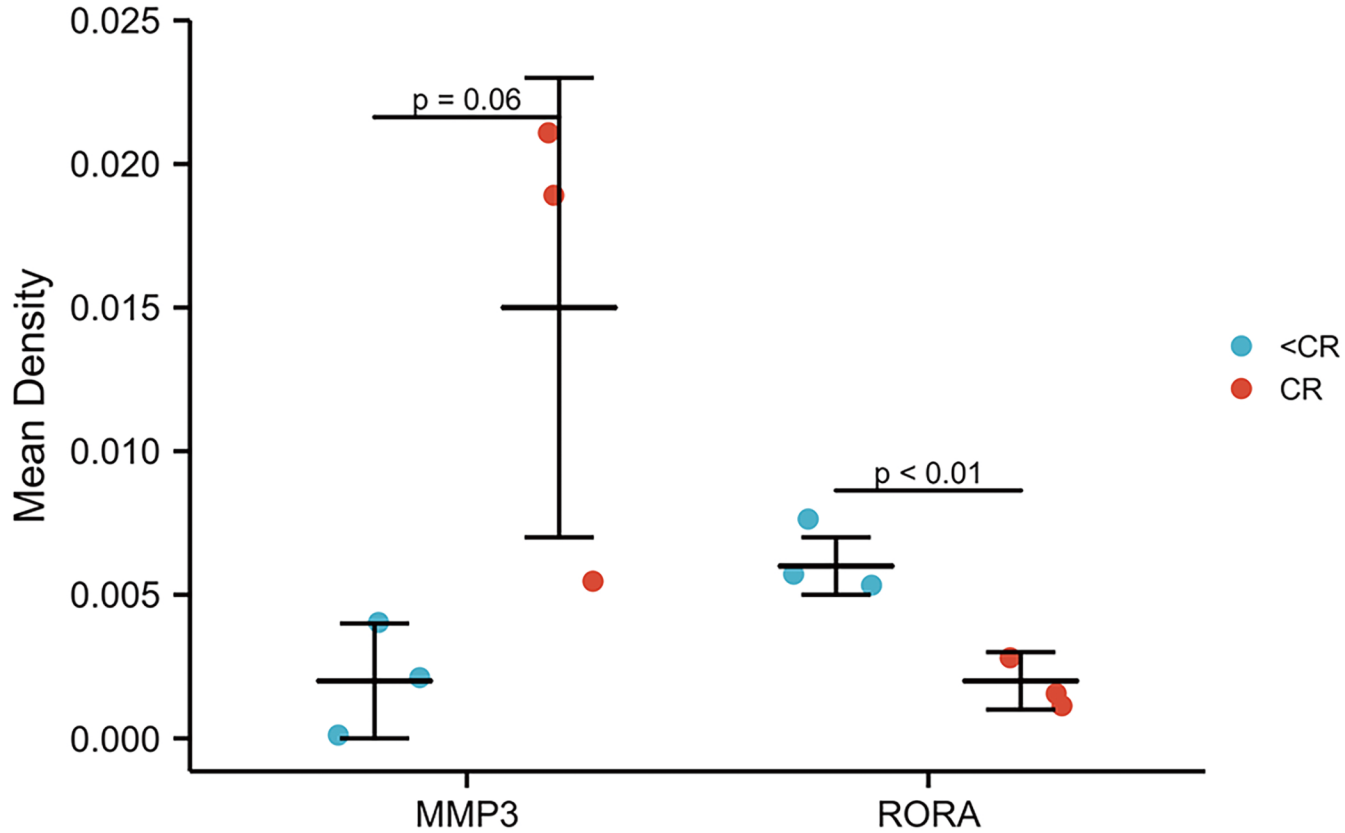

Supplement: Figure S5 — The CR group exhibited a higher mean density of MMP3 (marginally significant) and lower mean density of RORA (P < 0.01) compared to the < CR group. *P ≤ 0.05, **P ≤ 0.01. CR, complete remission; <CR, CR not achieved. [file peerj-11-15839-s005.pdf]
